# Pituitary adenylate cyclase-activating peptide induces neurite outgrowth in cultured monkey trigeminal ganglion cells: Involvement of receptor PAC1

**Published:** 2013-01-28

**Authors:** Emi Nakajima, Ryan D. Walkup, Atsuko Fujii, Thomas R. Shearer, Mitsuyoshi Azuma

**Affiliations:** 1 Senju Laboratory of Ocular Sciences, Senju Pharmaceutical Corporation Limited, Beaverton, OR; 2 Department of Integrative Biosciences, Oregon Health & Science University, Portland, OR

## Abstract

**Purpose:**

Our previous studies in the rabbit trigeminal nerve (TgN) showed that pituitary adenylate cyclase-activating peptide (PACAP) accelerated the extension of neuronal processes and recovery of corneal sensitivity. The purposes of the present study were 1) develop a procedure to culture trigeminal nerve (TgN) cells from monkeys, 2) test whether PACAP induces sprouting and elongation of axons in our culture system, 3) investigate the signaling mechanisms producing axon elongation induced by PACAP, and 4) test the action of PACAP on tear protein secretion by monkey lacrimal acinar cells.

**Methods:**

Primary cultures of TgN cells were established from rhesus monkeys. Cellular distribution of the PACAP receptor, PAC1, was determined with immunostaining. Axonal length in cultured TgN ganglion cells was evaluated with staining by antibody for neurofilament. mRNA expression was determined with quantitative real-time polymerase chain reaction (qPCR). Secretion of tear protein from cultured acinar cells was measured with immunoblotting.

**Results:**

Our results showed that dissociated, cultured TgN cells contained neuronal ganglion and Schwann cells, and the PAC1 receptor was expressed in both cell types. PACAP-27 significantly induced neurite outgrowth, which was inhibited by PACAP 6–27. Inhibitors for adenylate cyclase and phospholipase C also inhibited neurite outgrowth. Follistatin was upregulated by PACAP-27 during the culture period. PACAP enhanced secretion of tear proteins.

**Conclusions:**

Our data suggested PAC1 activation is involved in TgN neurite outgrowth.

## Introduction

Cornea, conjunctiva, lacrimal glands, eyelids, and meibomian glands form an integrated system that produces normal tear flow and the blink reflex. This system is regulated by sensory fibers (e.g., ophthalmic and lacrimal nerves) arising from the trigeminal nerve (TgN). Disruption of growth or regeneration of these TgN fibers leads to reduced tear flow, tear film instability, and dry eye [1].

Dry eye is a multifactorial disease characterized by ocular discomfort, visual disturbances, and potential erosion of the cornea. The prevalence of dry eye in the United States, Australia, and Asia ranges from 8% to 34% [2]. The underlying physiologic mechanism is believed to be a self-escalating cycle between tear film instability (e.g., excess evaporation) and tear film hyperosmolarity. This cycle can be activated by many factors, including anesthesia of the corneal-lacrimal gland reflex, aged-related decreased tear production, diabetes-associated neuropathy and microvascular changes, systemic and topical medications (beta-blockers and atropine-like drugs), autoimmune acinar damage in Sjögren syndrome, herpes/human immunodeficiency virus infections, and allergies.

A common cause of disruption of the TgN sensory fibers is laser-assisted in situ keratomileusis (LASIK). This widely accepted refractive surgery corrects myopia, and the number of patients undergoing LASIK surgery is increasing. However, the corneal flap created during LASIK immediately decreases the number of sub-basal and stromal nerve fiber bundles from the ophthalmic nerve by 90% [3]. Decreased corneal sensitivity may reduce reflex loop activity between the cornea and the lacrimal gland, cornea-induced blinking, and blink-induced meibomian gland secretion; all exacerbate dry eye. The treatments for dry eye include artificial tears, topical steroid or cyclosporine, hot compresses, punctal plugs, and autologous serum eye drops. Confocal microscopy revealed that intracorneal nerve fibers are regenerated within 3 to 6 months after LASIK surgery [3]. However, none of the current treatments for dry eye are targeted at regenerating the corneal sensory nerve.

Pituitary adenylate cyclase-activating peptide (PACAP) may induce regeneration of corneal sensory nerves. Our previous studies in rabbits showed that the shorter form corresponding to N-terminal 27 residues (PACAP-27) accelerated the extension of trigeminal neuronal processes and caused recovery of corneal sensitivity [4]. In undifferentiated cells, PACAP leads to neurite outgrowth and protection against neurotoxicity. PACAP immunoreactive nerve fibers have been identified in the central nervous system, TgN, and ocular tissues including the cornea [5].

PACAP is a well-conserved member of the vasoactive intestinal polypeptide (VIP)-glucagon-secretin superfamily. Active PACAP molecules include a 38 amino acid residue (PACAP-38), and PACAP-27 and PACAP-38 are post-translationally processed from a common precursor [6]. In the present studies in monkeys, we thus used PACAP-27 to compare the results to those in rabbits [6].

PACAP action on cells is mediated through G-protein-coupled receptors (GPCRs) from group II of the secretin receptor family. Three PACAP/VIP receptor genes have been identified; one encodes the PACAP-preferring receptor PAC1, whereas the other two encode receptors that respond equally to PACAP and VIP, VPAC1 and VPAC2. PAC1 not only activates a typical group II receptor signal cascade through adenylate cyclase (AC) [7] but is also coupled to the phospholipase C (PLC) pathway [8].

We tested the hypothesis that PACAP produced by the sensory fibers has two actions relevant to dry eye: 1) PACAP promotes neurite outgrowth from severed TgN processes and 2) stimulates tear protein secretion by lacrimal glands. Testing PACAP is best performed in human-relevant eye models, but culture of primary monkey trigeminal ganglion cells has not been characterized. Thus, the purposes of the present study were to 1) develop a procedure to culture TgN cells from monkeys, 2) test if PACAP induces sprouting and elongation of axons in our culture system, 3) investigate the signaling mechanisms producing axon elongation induced by PACAP, and 4) test the action of PACAP on tear protein secretion from monkey lacrimal acinar cells.

## Methods

### Experimental animals

Trigeminal nerves from rhesus monkeys (*Macaca mulatta*) were obtained at necropsy from the Oregon National Primate Research Center (Beaverton, OR) from experiments unrelated to the present studies. Experimental animals were handled in accordance with the Association for Research in Vision and Ophthalmology Statement for the Use of Animals in Ophthalmic and Vision Research and with the Guiding Principles in the Care and Use of Animals (DHEW Publication, NIH 80–23). Trigeminal nerves were obtained from 20 monkeys ranging in age from 0 to 15 years. The average time between death and dissection was less than 1 h.

### Monkey primary trigeminal nerve cell culture

The method for cell culture of monkey TgN was modified from Geenen’s method for porcine trigeminal nerves [9]. Fresh trigeminal ganglia from one monkey were dissected and cut into four longitudinal and cross-sectional pieces before enzymatic digestion was started with 0.2% collagenase A (Roche Applied Science, Indianapolis, IN). Every 30 min, dissociated cells were harvested by centrifugation at 200 ×*g* for 5 min, and the collagenase solution was reused for further digestion of TgN. After the ganglia were fully digested, the collected cell suspension was centrifuged at 200 ×*g* for 5 min. The pellet was resuspended in culture medium (Neurobasal Medium supplemented with 2% B-27 and 2 mM L-glutamine; Life Technologies, Grand Island, NY), and the cells were plated on polylysine and laminin-coated 24-well tissue culture plates (BD Bioscience, Franklin Lakes, NJ) at 2,500 neuron cells/cm^2^. Two trigeminal nerves from one monkey resulted in about 2.5×10^5^ TgN ganglion cells. One day after seeding, the cells were washed to remove non-adherent cells, and fresh medium was added. PACAP-27 (EMD Chemicals, Gibbstown, NJ), VIP (EMD Chemicals), and nerve growth factor (NGF; Sigma-Aldrich, St. Louis, MO) were used to induce neurite outgrowth. To investigate the mechanism of neurite outgrowth by PACAP-27, cells were treated with inhibitors 1 h before PACAP-27 or VIP was added. The N-terminus sequence of PACAP stimulates PACAP receptors, and the C-terminus sequence is involved in binding PACAP to receptors. Since deleting the N-terminal 5 amino acids inhibits PACAP-27-induced adenylate cyclase activation, PACAP6-27 (AnaSpec, Fremont, CA) was added to block the action of PACAP-27 [10,11]. PLC inhibitor U73122 (Sigma-Aldrich) and AC inhibitor 2’, 3′-dideoxyadenosine (ddA; Sigma-Aldrich) were used to determine downstream signal pathways induced by PACAP-27. Two days after the inducers were added, the cells were fixed for 30 min at room temperature in phosphate-buffered saline (PBS; Life Technologies) containing 10% formalin followed by immunocytochemistry. We did not observe obvious differences between tissues from young and adult monkeys during TgN cell culture or in axonal elongation studies.

### Immunohistochemistry of monkey trigeminal nerve sections

Trigeminal nerves were fixed in 10% formaldehyde and embedded in paraffin. Four-micron sections were blocked with 10% fetal bovine serum (FBS) in PBS for 30 min at room temperature. The sections were then incubated 2 h at room temperature with primary antibodies for neurofilament (NF; Sigma-Aldrich), glial fibrillary acidic protein (GFAP; Santa Cruz Biotechnology, Santa Cruz, CA), or PAC1 receptor (Abcam, Cambridge, MA). After the sections were rinsed with PBS, they were incubated with Alexa-Fluor 546-labeled antimouse immunoglobulin (IgG; Life Technologies) for NF, Alexa-Fluor 488- or 546-labeled antirabbit IgG (Life Technologies) for GFAP, Alexa-Fluor 488-labeled antimouse IgG (Life Technologies) for GFAP, and 1 μg 4’,6-Diamidino-2-phenylindole, dihydrochloride (DAPI; Life Technologies)/ml for nuclei staining for 1 h at room temperature. Negative controls were treated in the same manner, but normal rabbit or mouse IgG (Santa Cruz) was used in place of the primary antibody. All of the primary, secondary, and IgG antibodies were used at a dilution of 1:1,000. Immunoblotting on HeLa cell lysates with the antibody for the PAC1 receptor (Abcam, # ab54980) shows, along with the expected binding to the 50 kDa PAC1 receptor, a cross-reaction with an unknown 75 kDa band protein. Our mass spectrographic analysis identified the 75 kDa band as possibly human Lamin-A/C protein (data not shown). Lamin has been used as a nuclear membrane marker because the lamina matrix is located next to the inner nuclear membrane. Thus, DAPI nuclei staining overlapping green staining was interpreted as the location of cross-reacting lamin fibers. The optically sectioned fluorescence images of the stained samples were photographed using an inverted microscope (Axiovert 200), equipped with an AxioCam MRm camera and an ApoTome slider (Carl Zeiss Vision Gmbh, Hallbergmoos, Germany). Images were compiled in ImageJ 1.42 software (developed by Wayne Rasband, National Institutes of Health, Bethesda, MD) and Adobe Photoshop (Adobe Systems, San Jose, CA).

### Immunocytochemistry of monkey trigeminal nerve cells

Fixed cultured cells were blocked with 10% FBS in PBS for 30 min at room temperature. Fluorescent cell staining was performed as above. The confocal fluorescence images of stained samples were acquired using a Leica TCS SP laser scanning confocal microscope with an inverted Leica IRBE (Leica Microsystems, Heidelberg, Germany) with an HCX PL APO 40.0×1.25 OIL UV objective (NA 1.25 and zoom 2.0); with an HCX PL APO 40.0×1.25 OIL UV objective (NA 1.25 and zoom 1.0); with an HCX PL APO 40.0×1.25 OIL UV objective (NA 1.25 and zoom 2.0); and with a PL APO 100.0×1.40 OIL UV objective (NA 1.4 and zoom 3.0). The 361-nm line of a UV argon laser was used to excite DAPI, the 488-nm line of an argon laser to excite Alexa-Fluor 488, and the 568-nm line of a krypton laser to excite Alexa-546. Images were compiled in ImageJ and Adobe Photoshop.

### RNA extraction and reverse transcription

Total RNA from dissociated monkey trigeminal nerve cells, lacrimal acinar cells, and cultured trigeminal cells treated with PACAP was extracted using TRIzol reagent (Life Technologies) following the manufacturer’s instructions. After phase separation, RNA was precipitated with 75% ethyl alcohol, bound to a glass fiber filter, washed, and collected using an RNA extraction kit (RNEasy Plus Universal Mini Kit; Qiagen, Valencia, CA). RNA quality was determined on a 2100 Bioanalyzer (Agilent Technologies, Palo Alto, CA). Concentration of total RNA was determined with RiboGreen reagent (Life Technologies). RNA was reverse transcribed at a concentration of 50 ng/μl with 6 U/μl SuperScript II Reverse Transcriptase following the manufacturer’s instructions with 0.25 U SUPERase-In/μl, 12.5 ng random primers/μl, and 0.5 mM deoxynucleotide triphosphates (dNTPs; Life Technologies). No DNase reaction was performed since all probes were designed to cross exon boundaries.

### Quantitative real-time polymerase chain reaction

Custom primers and probes for Rhesus macaque PAC1, VPAC1, and VPAC2 were developed by Life Technologies using the Custom TaqMan Gene Expression Assays (Table 1). Assays for follistatin (Rh01121161_m1) were performed with the TaqMan Gene Expression Assay (Life Technologies). The quantitative real-time polymerase chain reaction (qPCR) probe for the PAC1 gene aligns to all four known transcript variants. All assays contained 250 nM probe, 900 nM each primer, 1X PCR Master Mix (Life Technologies), and 50 ng cDNA in 20 µl total volume. Following initial uracil DNA glycosylase (UDG) activation at 50 °C for 2 min and 95 °C for 10 min, PCR reactions were run for 40 cycles at 95 °C for 15 s and 60 °C for 1 min. Fluorescence measurements were taken at each cycle following extension (StepOnePlus, Life Technologies).

**Table 1:** Primers for quantitative PCR.

Genes	Primers (5′-3′)
*PAC1*	F: GTGGCTGTTCTCTACTGTTTTCTGA
	R: GCTTCGCCATTTTCGCTTGATC
	Probe: TCCGCTTGTACCTCGCC
*VPAC1*	F: CCCCTCATCTTCAAGCTCTTCTC
	R: CAGGTGTGTCCAACCTTCGT
	Probe: CACATTGCGGCCTTGAAT
*VPAC2*	F: CTCTGATGTCTCTTGCAACAGGAA
	R: CAGGAACAGGTTCAGATGGATGTAA
	Probe: CCTCTTCAGGAAGCTGC
*GAPDH*	F: TGCACCACCAACTGCTTA
	R: CATGAGTCCTTCCACGATACCAA
	Probe: CCCTGGCCAAGGTCATCCATGA
*HPRT1*	F: TCCATTCCTATGACTGTAGATTTTATCAGACT
	R: AGTTGAGAGATCATCTCCTCCGATT
	Probe: CCTGTTGACTGGTCATTACA

Quantification of PACAP receptor gene transcripts in the trigeminal nerve was determined using an absolute method relative to a standard dilution of synthetic oligonucleotides. PCR efficiencies and limit of detection (LoD) for each assay were determined from standard curves. LoDs varied among genes for PAC1, VPAC1, and VPAC2 at 8.81, 232, and 343 copies per 20 μL reaction, respectively, due to variances in qPCR efficiencies and stochastic effects between different primer sets when used for measuring low copy numbers. Normalized expression to glyceraldehyde 3-phosphate dehydrogenase (GAPDH) and hypoxanthine phosphoribosyltransferase 1 (HPRT1) was converted to absolute copy numbers by comparison to a standard curve made from serial dilutions of a quantified (DNAQuant), polyacrylamide gel electrophoresis–purified oligomer. Relative ΔΔG expression analysis was used for follistatin qPCR. Transcript levels were well within the LoD and normalized by the geometric mean for the GAPDH and HPRT1 expression levels. Sequences for the primers and probes for GAPDH and HPRT1 are shown in Table 1.

### Cell counting and statistical analysis

For quantitative measurement of neurite outgrowth, the total cell numbers of neurofilament stained cells were counted in each well (10–17 areas, 3.35×2.50 mm) as described previously [4]. Briefly, a cell with a neurite was defined as a cell with a neurofilament-stained cell body and a process extending at least twice the diameter of the cell body. Data are expressed as the percentage of cells with a neurite compared to the total stained cells.

Statistical analyses of the data were performed with Dunnett’s *t* test (JMP 8.0.1 software; SAS Institute, Cary, NC). p<0.05 was considered statistically significant.

### Monkey lacrimal acinar cell culture and measurement of secreted tear proteins

Lacrimal acinar cells were isolated as described previously [12]. Briefly, lacrimal glands isolated from rhesus monkeys were rinsed in DMEM/F12 (Life Technologies) containing 0.1 mg soybean trypsin inhibitor (STI; Sigma-Aldrich) /ml and then minced with scissors. After centrifugation, cells were obtained by dissociation. Hanks balanced salt solution (HBSS; Life Technologies) containing 0.76 mg/ml EDTA was added and incubated for 15 min to activate trypsin. The fragments were collected by centrifugation and then incubated for 15 min in DMEM/F12 containing 200 U/ml collagenase (Roche Applied Science), 698 U/ml hyaluronidase (Worthington Biochemical Corporation, Lakewood, NJ) and 10 U/ml DNase I (Roche Applied Science) (CHD). The dissociation of cells was repeated one more time, and then DMEM/F12 containing 20% FBS was added to stop the enzyme reaction. Dissociated cells were obtained by filtration using 100 μm and 40 μm nylon meshes, and the cells were segregated by centrifugation with a discontinuous 10, 30, and 60% Percoll gradient (Sigma-Aldrich). Acinar cells were collected from the 30-60% interface. The cells were plated at 1×10^5^ cells/cm^2^ on rat collagen I (0.01 mg/cm^2^; BD Bioscience) coated plates with the culture medium (DMEM/F12; Life Technologies) supplemented with 10 ng dexamethasone (Sigma-Aldrich)/ml, 50 ng epidermal growth factor (Life Technologies)/ml, 10 μg glutathione (Sigma-Aldrich)/ml, 1X ITS (Sigma-Aldrich), 25 μg L-ascorbic acid (Sigma-Aldrich)/ml, 1 mM putrescine (Sigma-Aldrich), and 50 μg gentamicin (Life Technologies)/ml. After overnight culture, the acinar cells were rinsed, pretreated with supplement-free medium for 30 min, and then incubated with PACAP-27 or VIP for 15 min at 37 °C. After 15 min induction, the cultured media were collected, and detached cells were removed with centrifugation. The collected media were concentrated and subjected to immunoblotting.

### Immunoblotting for secreted tear proteins from cultured monkey lacrimal acinar cells

The concentrated media were separated by 4%–12% NuPAGE Bis-Tris gels with 2-(N-morpholino)ethanesulfonic acid (MES) buffer (Life Technologies). The gels were then electrotransferred to polyvinylidene fluoride membranes (EMD Millipore, Billerica, MA) for immunoblotting. Membranes were blocked with 0.5% skim milk in Tris-buffered saline with 0.05% Tween-20 (TTBS) for 30 min at room temperature, and incubated with primary antibodies for lactoferrin (1:5,000 dilution, Sigma-Aldrich) in 1% bovine serum albumin in TTBS overnight at 4 °C. Membranes were then rinsed in TTBS and incubated for 1 h at room temperature with horseradish peroxide-conjugated goat antirabbit secondary antibody (1:10,000–5,000, Santa Cruz). Membranes were developed with chemiluminescence (ECL Plus; GE Healthcare BioSciences, Piscataway, NJ), and images were captured with a FluorChem FC2 imager (ProteinSimple, Santa Clara, CA). Band intensities were measured with ImageJ. Statistical analyses of the data were performed as described above.

## Results

### Monkey trigeminal nerve cell culture

**Figure 1:**
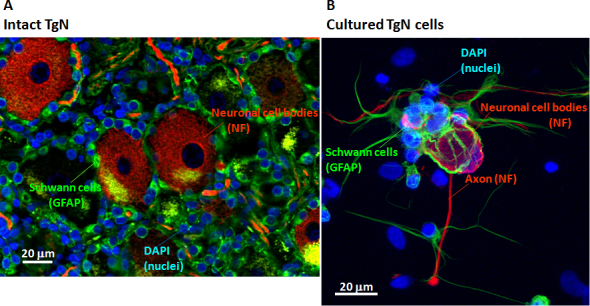
Immunohistologic images of monkey trigeminal nerve (TgN) tissue and cultured cells are shown. **A**: Fresh, whole monkey trigeminal nerve ganglion was triple-labeled with antibodies to the following cell markers: green Schwann cells (GFAP) surrounding red TgN ganglion cells (neurofilament) and blue nuclei (DAPI). **B**: Cultured monkey trigeminal ganglion nerve cells after 3 days were triple-labeled with antibodies to the cell markers listed above.

Immunohistochemistry of whole monkey TgN showed ganglion cells (red) surrounded by glial cells (Schwann cells, green; Figure 1A). After dissociation, primary culture of the monkey TgN cells was established and similarly showed ganglion cells (red) and Schwann cells (green; Figure 1B). In these unstimulated cells at 3 days, extended axons from soma were only occasionally observed. This TgN culture also contained unknown cells that were negative to neurofilament, glial-fibrillary acidic protein, and fibroblast staining (data not shown). The mean cell proportions were TgN ganglion cells 3.5%, Schwann cells 76.1%, and unknown cells 20.4%.

### Pituitary adenylate cyclase-activating peptide with 27 residues enhanced neurite outgrowth in cultured trigeminal nerve cells

**Figure 2:**
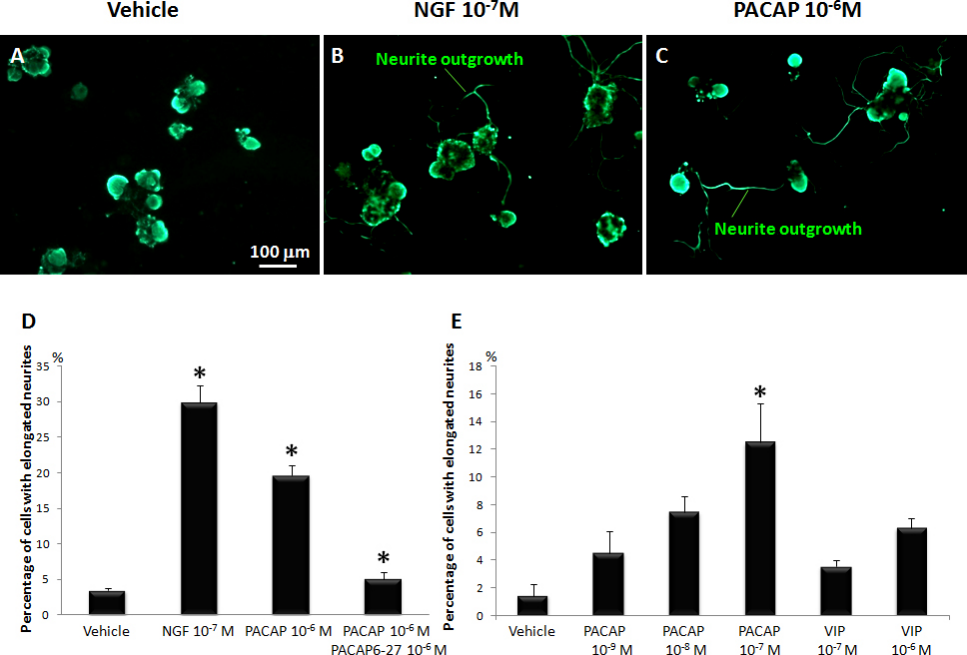
Treatment with 27-residue pituitary adenylate cyclase-activating peptide (PACAP-27) enhanced neurite outgrowth in trigeminal nerve (TgN) cells. **A**: Cultured vehicle TgN cells were labeled with immunofluorescent antibody to neurofilament and treated with **B**: 10^-7^ M nerve growth factor (NGF) or C: 10^-6^ M PACAP. **D**: The graph shows percentage of TgN cells with elongated neurites when incubated with NGF, PACAP-27, or PACAP-27 plus inhibitor PACAP 6-27. Data are means±SEM (n=9). *p<0.05 is relative to vehicle for NGF and PACAP, or relative to PACAP for the PACAP plus PACAP antagonist group (Dunnett’s t test). **E**: The graph shows percentage of TgN cells with elongated neurites after stimulation with increasing dosages of PACAP-27 or control VIP. Data are means±SEM (n=3). *p<0.05 is relative to vehicle.

PACAP-27 and positive control NGF enhanced neurite outgrowth in cultured TgN ganglion cells (Figures 2A-D). The effect of PACAP-27 on elongation was quantified and compared to VIP, a well-studied and homologous signal transduction peptide. PACAP-27 and VIP (Figure 2E) increased neurite outgrowth in a dose-dependent manner, but VIP required 100-fold higher concentrations than PACAP-27. Receptor activation by PACAP-27 in rat superior cervical ganglion cells was reported to be between 10^−9^ and 10^−8^ M [13]. However, our results showed that 10^−7^ to 10^−6^ M PACAP-27 was required to cause statistically significant neurite outgrowth. PACAP showed a trend toward dose-dependent increase of neurite outgrowth at 10^−9^ M to 10^−8^ M, but this was not statistically significant. (Our impression was that the total TgN ganglion cell numbers, although not quantified, were not affected by treatment with NGF, PACAP-27, or VIP.)

### Enhancement of neurite outgrowth by pituitary adenylate cyclase-activating peptide with 27 residues through the PAC1 receptor

**Figure 3:**
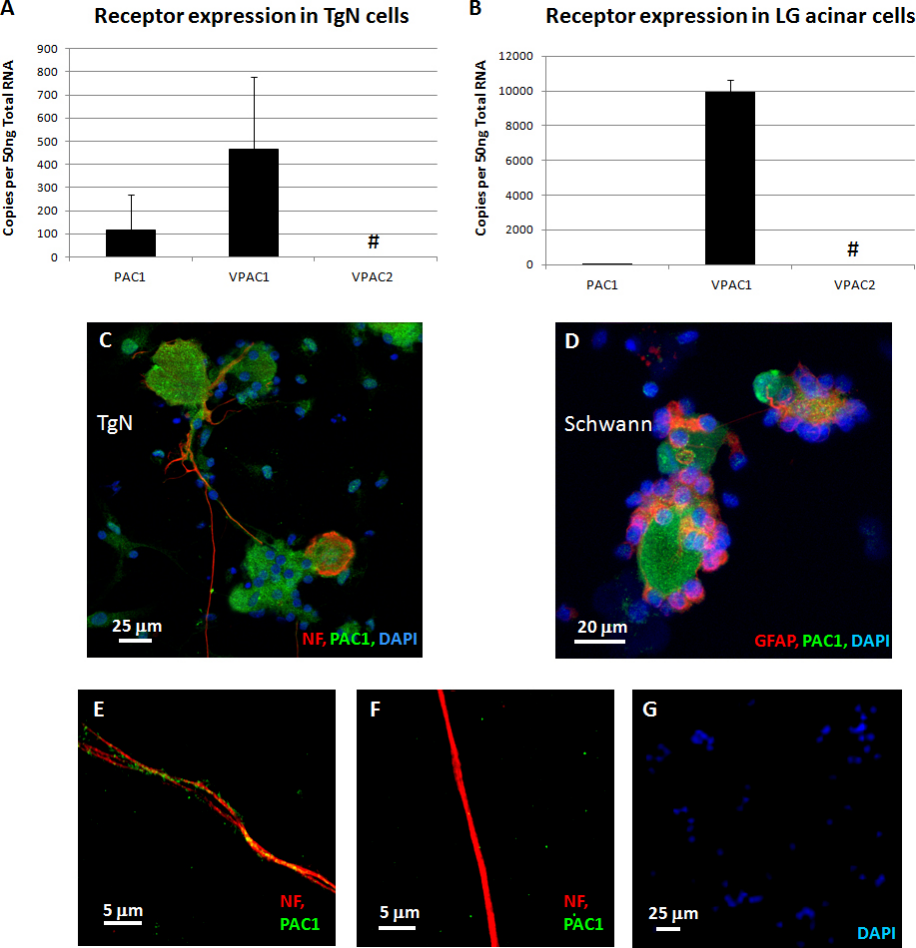
Pituitary adenylate cyclase-activating peptide (PACAP) binding receptors were expressed in trigeminal nerve (TgN) cells and lacrimal gland (LG) acinar cells. **A**: Total mRNA extracted from dissociated TgN cells showed transcripts for the three PACAP receptors. Data are means±SD (n=6). #Amplification was detected, but it was below the linear limit of detection (LoD). **B**: Total mRNA extracted from dissociated lacrimal gland (LG) acinar cells also showed transcripts for PACAP receptors. Data are means±SD (n=4). Positive immunohistochemical staining for PAC1 receptors appeared orange in **C**: TgN ganglion cells and in **D**: Schwann cells. This was due to the green PAC1 receptor staining overlaying the red cell-specific staining. **E**: Higher power magnification showed green dot staining for PAC1 receptors over the red axons. Where blue-staining by 4',6-diamidino-2-phenylindole, dihydrochloride (DAPI)-nuclei overlaid green areas, this was considered positive staining for the nuclear membrane lamin; while other green areas without nuclei were considered PAC1 receptor-positive (see Materials and Methods). **F**: Higher power magnification showed that the green PAC1 receptor staining was eliminated by peptide neutralization of the primary antibody. **G**: When primary antibodies for NF and PAC1 were eliminated, only blue-staining (DAPI) nuclei were observed.

Note that the receptor antagonist PACAP6-27 [10,11] almost completely inhibited PACAP-27-induced neurite outgrowth (Figure 2D). Further, all three PACAP binding receptors, PAC1, VPAC1, and VPAC2, were expressed in TgN cells from monkeys, but at fairly low levels (Figure 3A). For comparison, VPAC1 receptor expression was 20-fold higher in cultured monkey lacrimal gland (LG) acinar cells than in TgN cells, while PAC1 and VPAC2 receptor expression levels were at or below detection limits (Figure 3B).

Immunocytostaining was positive for the PAC1 receptor in TgN ganglion (Figure 3C) and Schwann cells (Figure 3D). Two staining patterns were observed: 1) orange resulting from the green PAC1 receptor staining overlaying the red cell marker protein staining and 2) blue DAPI nuclei staining overlapping green protein staining, interpreted as probable staining for nuclear lamin-A/C proteins (see Materials and Methods).

At higher magnification (Figure 3E), PAC1 receptor-positive staining was observed as green dots on the red TgN neuronal axons. Staining for both bands was blocked when the anti-PACAP receptor antibody was neutralized with a peptide epitope based on the N-terminal 50 amino acids of the PACAP receptor (GenWay Biotech, San Diego, CA; data not shown). When the PAC1 receptor antibody was preabsorbed with the receptor peptide, all staining on the neuronal body, axons, and Schwann cells was eliminated (Figure 3F). Thus, except the nuclei, the green dot staining indicated the presence of the PAC1 receptor on neuronal bodies, axons, and Schwann cells. Positive staining for the NF and the PAC1 receptor was eliminated when the primary antibodies for the NF and the PAC1 receptor were omitted (Figure 3G).

### Downstream pathways in pituitary adenylate cyclase-activating peptide–induced neurite outgrowth

**Figure 4:**
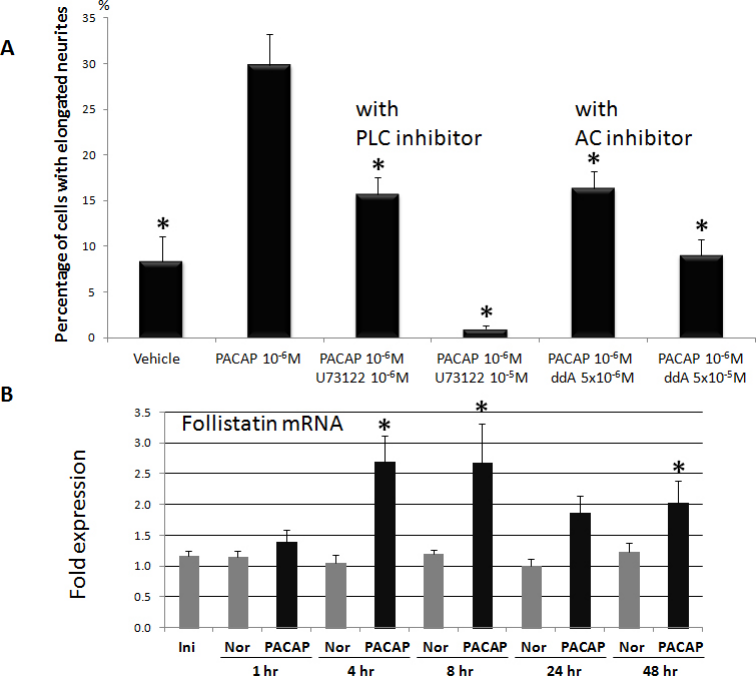
Pre-treatment with inhibitors of pathways, that are downstream of the action by pituitary adenylate cyclase-activating peptide (PACAP), attenuated PACAP induced neurite outgrowth. **A**: The graph shows percentage of cells with elongated neurites in trigeminal nerve (TgN) cells stimulated with PACAP alone or with downstream pathway inhibitors U73122 (phospholipase C [PLC] inhibitor) or ddA (adenylate cyclase inhibitor). Data are means±SEM (n=3). *p<0.05 is relative to vehicle (Dunnett’s t test). **B**: Total mRNA extracted from TgN cells treated with PACAP-27 (black columns) showed higher follistatin expression over vehicle (Nor, gray columns). Initial sample (Ini) was collected immediately before addition of PACAP. Data are means±SD (n=3). *p<0.05 is relative to vehicle (Student t test).

The PAC1 receptor activates AC and PLC-dependent signaling cascades via G proteins [7,8]. In our monkey TgN cells, PACAP-induced neurite outgrowth was significantly inhibited by the PLC inhibitor (U73122) and by the AC inhibitor (ddA) in a dose-dependent manner (Figure 4A). Higher concentrations of ddA or U73122 alone completely inhibited neurite outgrowth.

Activation of AC and PLC pathways by PACAP could also upregulate other relevant genes. In our cultured monkey TgN cells, PACAP-27 upregulated expression of mRNA for the neuronal differentiation gene, follistatin, which showed statistically significant increases at 4 and 8 h and then decreased gradually (Figure 4B).

### Effect of pituitary adenylate cyclase-activating peptide on tear protein secretion

**Figure 5:**
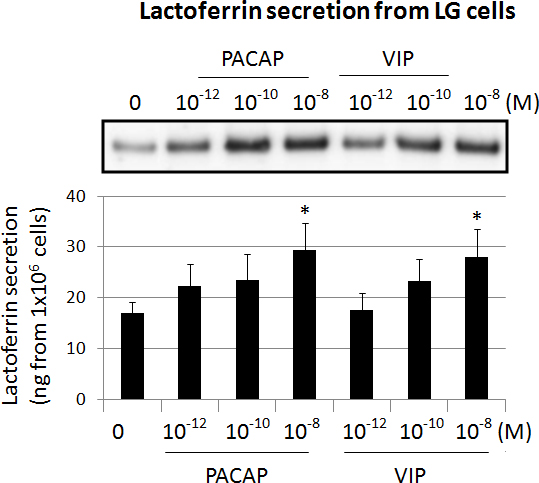
Amounts of lactoferrin protein secreted into the medium from cultured lacrimal gland (LG) acinar cells were analyzed by immunoblotting. Cells were treated with PACAP-27 and VIP at increasing concentrations. Below: The graph shows density of lactoferrin protein bands, expressed as mean±SEM (n=6). *p<0.05 is relative to vehicle with Dunnett’s t test.

As described above, neuronal stimulation of the cornea is coupled to lacrimal gland tear flow. We next used lacrimal acinar cells, where the VPAC1 receptor is dominant, to evaluate if there is a difference between PACAP-27 and VIP in the ability to induce tear protein secretion. In LG acinar cells, in contrast to TgN, PACAP-27 and VIP showed similar, dose-dependent stimulation of tear protein secretion (lactoferrin) into the medium (Figure 5).

## Discussion

### Characteristics of monkey trigeminal nerve cells in culture

Culture of adult monkey trigeminal ganglion neurons was first reported in 1993 [14], and the present study provided further characterization of the TgN ganglion and Schwann cells in such cultures. Neuronal cells cultured for 3 days possess axons (Figure 1B, red), which innervate the cornea in vivo. PACAP induced monkey trigeminal cells to produce elongated axons (Figure 2). The ganglion cell bodies were 20 to 30 times larger than the Schwann cells that surrounded the ganglion. Schwann cells also formed fiber-like structures (Figure 1B, green) extending to the neuronal cell bodies and axons. This may mimic myelination by Schwann cells in vivo, and emphasizes the relevance of this monkey model for testing possible human medications such as PACAP-27.

### Mechanism of pituitary adenylate cyclase-activating peptide–induced neurite outgrowth through the PAC1 receptor

Although PAC1 expression was less than VPAC1, PAC1 is probably most directly responsible for PACAP-induced neurite outgrowth because of the following: 1) Outgrowth was inhibited by the inhibitor of PACAP-27, PACAP6-27 [10,11] (Figure 2D). 2) PAC1 displays two to three orders of magnitude greater affinity for PACAP than for VIP [15]. 3) In our experiments, the bioactivity of PACAP-27 toward neurite outgrowth in TgN cells had a 100-fold stronger effect than VIP. 4) High concentrations of the VPAC2 receptor-specific agonist, Ro 25–1553, caused only minimal neurite outgrowth in human SH-SY5Y neuroblastoma cells after 4 days of culture [16], suggesting that VPAC receptors are not major factors for neurite outgrowth. 5) In contrast to TgN cells, PACAP-27 and VIP stimulated tear lactoferrin secretion at the same rate in LG acinar cells (Figure 5), where VPAC1 receptor expression was dominant. 6) The PAC1 receptor was expressed not only on the cultured TgN cell bodies but also on the neuronal axons themselves (Figure 3). These data suggested PACAP binding to PAC1 may occur directly on peripheral neuronal axons in the cornea to stimulate neurite outgrowth.

### Activation of phospholipase C/protein kinase C and adenylate cyclase/protein kinase A pathways by pituitary adenylate cyclase-activating peptide with 27 residues through PAC1 during neurite outgrowth

PAC1 is coupled to AC and PLC, which induce production of cyclic adenosine monophosphate (cAMP) and increase intracellular Ca^2+^ subsequent to activation of protein kinase A (PKA) and protein kinase C (PKC) [7,8]. In our present studies, PLC inhibitor U73122 and AC inhibitor ddA completely inhibited PACAP-induced neurite outgrowth (Figure 4A). This indicates cross talk between the AC/PKA and PLC/PKC pathways during PACAP-induced neurite outgrowth. Note also that with 10^−5^ M U73122, neurite outgrowth was inhibited more than in the vehicle group (Figure 4A), suggesting that normal physiologic neurite outgrowth may be regulated via the PLC pathway. These conclusions could be further tested by axonal elongation studies with PKA and mitogen-activated protein kinase inhibitors using the present monkey model.

The AC/PKA pathway might also cause neurite growth by influencing the expression of other genes. We found that the expression level of the neuronal differentiation gene follistatin was upregulated by PACAP-27 throughout the culture period (Figure 4B). Follistatin was also upregulated 3.37 fold in chicken TgN cells cultured with PACAP-27 for 15 min [17]. Follistatin stimulated neurite outgrowth of the neuroblastoma cell line IMR-32 [18]. These data suggested that PACAP-27-induced follistatin expression may be one of the mechanisms stimulating neurite outgrowth in monkey TgN cells due to activation of the AC/PKA and PLC/PKC pathways confirmed by PACAP-induced TgN neurite outgrowth.

### Stimulation of tear protein secretion

In addition to neurite outgrowth, PACAP-induced protein secretion from monkey lacrimal acinar cells was observed (Figure 5). PACAP null mice showed dry eye-like symptoms, including tear reduction and corneal keratinization [19]. Topical administration of PACAP stimulated tear secretion via the cAMP/PKA cascade. In lacrimal acinar cells, PACAP also stimulated translocation of the water transporter, aquaporin 5 (AQP5), from the cytosol to the membrane and induced tear secretion [19]. Thus, an additional advantage of PACAP may be its ability to promote tear protein secretion from lacrimal gland cells through the VPAC1 receptor.

### Conclusion

These studies in a novel, relevant monkey model suggest that treatment of dry eye with PACAP-27 may be beneficial because of its stimulating effect on TgN neurite outgrowth to the cornea and because PACAP-27 stimulates tear protein secretion from the lacrimal gland.
